# A Prompt-Preserving SAM ViT-B Adaptation Framework with Historical Branch Fusion and Soft Convolutional Expert Weighting for Medical Image Segmentation

**DOI:** 10.3390/bioengineering13080914

**Published:** 2026-08-13

**Authors:** Shengyang Ping, Zhijie Lin, Liliang Lin, Lei Zhao, Lisha Ye, Bangguo Wang, Tao Wang

**Affiliations:** 1 School of Computer Science and Technology, Zhejiang University of Science and Technology, Hangzhou 310023, China; 2 College of Computer Science and Technology, Zhejiang University, Hangzhou 310027, China; 3 Sir Run Run Shaw Hospital, Zhejiang University School of Medicine, Hangzhou 310016, China

**Keywords:** medical image segmentation, Segment Anything Model, SAM ViT-B, prompt robustness, historical branch fusion, convolutional experts, parameter-efficient fine-tuning

## Abstract

Promptable medical segmentation models can be sensitive to small targets, weak boundaries, heterogeneous texture, and inaccurate user prompts. We propose HBF-BCER, a prompt-preserving adaptation of the SAM ViT-B architecture for medical image segmentation. Historical Branch Fusion (HBF) reinjects aligned bottleneck summaries from previous enhanced encoder blocks, while Balanced Convolutional Expert Routing (BCER) applies load-balanced, pixel-wise soft weighting to heterogeneous convolutional experts. The evaluation uses fixed held-out evaluation sets, three training seeds, matched original SAM ViT-B, image-encoder and mask-decoder full fine-tuning, decoder-adapter, LoRA, and DoRA baselines, repeated prompt perturbations, paired statistics, and internal module ablations. HBF-BCER achieved Dice scores of 96.69±0.12% and 92.24±0.18% for REFUGE2 disc and cup, 96.18±0.31% for ISIC2016, and 92.08±0.19% for TNMIX. Image-encoder and mask-decoder full fine-tuning produced slightly higher Dice for the REFUGE2 disc and TNMIX, whereas HBF-BCER produced higher Dice for the REFUGE2 cup and ISIC2016 and the lowest HD95 on all four targets. Among the four paired comparisons with available per-image outputs, HBF-BCER showed significant gains for REFUGE2 cup versus SAM + Adapter and LoRA and for TNMIX versus SAM + Adapter; the ISIC2016 difference versus full fine-tuning was not significant. The method reduced trainable parameters by 81.5% relative to image-encoder and mask-decoder full fine-tuning but increased total parameters, FLOPs, and inference latency. These results support HBF-BCER as a parameter-efficient training strategy rather than a generally lightweight or uniformly superior model.

## 1. Introduction

Medical image segmentation is a central component of computer-assisted diagnosis, treatment planning, and quantitative image analysis. Promptable foundation models provide an attractive alternative to fully automatic segmentation because a sparse point or box can identify the intended anatomical structure or lesion. The Segment Anything Model (SAM) established the image-encoder–prompt-encoder–mask-decoder paradigm [1], and MedSAM adapted this paradigm to medical images through large-scale medical-domain training [2]. Nevertheless, prompt-conditioned segmentation remains difficult when targets are small, boundaries are weak, appearances are heterogeneous, or acquisition noise is substantial.

These limitations are evident in the three two-dimensional tasks considered in this work. REFUGE2 contains nested optic disc and cup structures, so small boundary errors can disproportionately affect cup segmentation [3,4]. ISIC2016 lesions have irregular and visually ambiguous borders [5,6]. Thyroid ultrasound images contain speckle noise, low contrast, and uncertain nodule margins [7,8,9]. In each case, the decoder depends on image tokens that must retain early structural evidence while remaining responsive to the prompt.

Recent work has improved medical segmentation through task-specific detection and cascaded processing. For example, Nefediev et al. used prostate detection followed by cascaded segmentation on T2-weighted MRI [10]. More directly related to promptable segmentation, Gu et al. improved SAM-based lesion segmentation through feature resampling, adaptive weighting, and multilevel fusion under prompts of varying quality [11]. These studies demonstrate the value of structural priors and multilevel feature reuse. HBF-BCER addresses a different design point: it retains the original point/box interface and introduces cross-layer historical information and soft convolutional expert weighting inside selected image-encoder blocks rather than using a task-specific detection cascade or a decoder-side multilevel fusion module.

We propose HBF-BCER, a prompt-preserving adaptation of the SAM ViT-B architecture for medical image segmentation. Historical Branch Fusion (HBF) maintains a short memory of bottleneck features from previous enhanced blocks and reinjects their aligned summary into deeper blocks. Balanced Convolutional Expert Routing (BCER) applies pixel-wise soft weighting to heterogeneous convolutional experts in bottleneck space while discouraging expert collapse. A lightweight decoder adapter is held constant across adapter-based comparisons. The evaluation uses fixed held-out sets, three independent training seeds, matched LoRA and DoRA baselines, repeated prompt perturbations, paired statistical tests, and internal module ablations.

The main contributions are as follows:

- We introduce a prompt-preserving encoder adaptation that combines cross-layer historical memory with spatially varying soft convolutional expert weighting.
- We distinguish the effects of the original SAM ViT-B checkpoint, image-encoder and mask-decoder fine-tuning, a decoder adapter, LoRA, DoRA, HBF alone, BCER alone, and the complete HBF-BCER model under the same data and prompt protocol.
- We evaluate prompt robustness using accurate, random, boundary-near, shifted-point, and box-jitter conditions with repeated prompt sampling.
- We report mean ± standard deviation across training seeds, paired bootstrap confidence intervals, Wilcoxon signed-rank tests with Holm correction, internal HBF/BCER ablations, and enlarged overlay/error-map visualizations.

## 2. Related Work

### 2.1. Promptable Medical Image Segmentation and Prompt Robustness

SAM combines a high-capacity image encoder, a prompt encoder, and a lightweight mask decoder [1]. MedSAM [2], SAM-Med2D [12], and Medical SAM Adapter [13] adapt this interface to medical images. Independent evaluations have shown that SAM-style models behave unevenly across modalities, target sizes, and prompt types [14,15]. Robust lesion segmentation under inaccurate prompts has therefore become an important problem. Gu et al. [11] use feature resampling, adaptive weighting, and multilevel fusion to improve prompt robustness. In contrast, HBF-BCER modifies selected encoder blocks and leaves the prompt encoder and user-facing interaction unchanged.

Task-specific cascaded approaches provide a complementary perspective. Nefediev et al. [10] first localize the prostate region and then apply cascaded segmentation networks. Such a pipeline benefits from explicit detection guidance but is tailored to a specific anatomy. HBF-BCER does not introduce a detector; it aims to preserve general promptability across fundus, dermoscopy, and ultrasound tasks.

### 2.2. Structural Preservation and Historical Feature Reuse

U-Net and UNet++ reuse multilevel encoder information through skip pathways [16,17], while nnU-Net demonstrates the value of carefully controlled training pipelines [18]. Transformer-based methods such as TransUNet and multiresolution variants emphasize long-range context and scale-aware structure [19,20]. HBF is motivated by structural reuse but differs from conventional encoder–decoder skip connections. It stores compressed representations from previous enhanced ViT blocks and adds an aligned historical bias inside the encoder, before prompt-conditioned decoding.

### 2.3. Parameter-Efficient Adaptation and Expert Routing

Adapters and low-rank updates reduce the number of trainable parameters required for foundation-model adaptation [21,22]. LoRA freezes pretrained weights and learns low-rank update matrices, while DoRA decomposes weight magnitude and direction and applies low-rank adaptation to the directional component [23]. These methods are strong parameter-efficient fine-tuning (PEFT) baselines but do not explicitly encode historical cross-layer memory or spatially varying convolutional receptive fields.

Mixture-of-experts mechanisms allocate different inputs to specialized branches [24]. Medical segmentation studies have explored attention-based experts, MoE-enhanced SAM, and shape experts [25,26,27]. BCER differs from sparse top-*k* routing: all experts participate through soft weights at every bottleneck pixel. The load-balancing term discourages routing collapse but does not make the model computationally sparse.

## 3. Materials and Methods

### 3.1. Overall Framework

**Figure 1:**
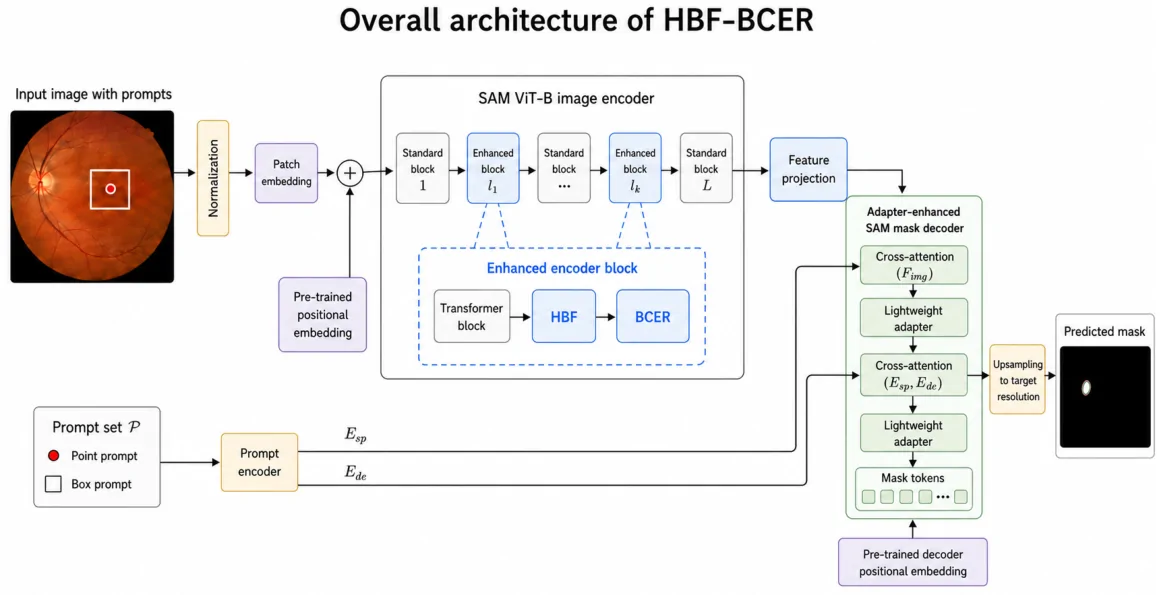
Overall architecture of HBF-BCER. Selected SAM ViT-B image-encoder blocks are enhanced by Historical Branch Fusion (HBF) and Balanced Convolutional Expert Routing (BCER). The point/box prompt interface is unchanged, and the same decoder-adapter design is used for adapter-based controls. Ellipses indicate omitted intermediate encoder blocks or repeated mask tokens for compact visualization.

Figure 1 summarizes the framework. Given a normalized image $\tilde{I}\in R^{B\times 3\times H\times W}$ and prompt set *P*, patch tokens are combined with the interpolated pretrained positional embedding:

(1) $$X^{\left(0\right)}=\phi_{\mathrm{patch}}\left(\tilde{I}\right)+I\left(E_{\mathrm{pos}}^{\mathrm{enc},\mathrm{pre}}\right).$$

For an unmodified image-encoder layer *l*,

(2) $$X^{\left(l+1\right)}=T_{l}\left(X^{\left(l\right)}\right).$$

For selected enhanced layers $L_{\mathrm{enh}}=\left\{3,6,9,12\right\}$ (one-based indexing), the transformer output is refined sequentially by HBF and BCER:

(3) $$\begin{array}{cc} Y_{t} & =T_{l_{t}}\left(X^{\left(l_{t}\right)}\right), \end{array}$$

(4) $$\begin{array}{cc} {\bar{X}}_{t} & =Y_{t}+B_{\mathrm{hbf}}^{\left(l_{t}\right)}+B_{\mathrm{hist}}^{\left(l_{t}\right)}, \end{array}$$

(5) $$\begin{array}{cc} X^{\left(l_{t}+1\right)} & ={\bar{X}}_{t}+B_{\exp}^{\left(l_{t}\right)}. \end{array}$$

The final image embedding is $F_{\mathrm{img}}=\phi_{\mathrm{proj}}\left(X^{\left(L\right)}\right)$. The frozen prompt encoder produces sparse and dense prompt embeddings, and an adapter-enhanced mask decoder predicts low-resolution masks that are upsampled to the target resolution. HBF and BCER modify only the image representation; prompt coordinates and prompt semantics are unchanged.

### 3.2. Adaptation Scope and Baseline Definitions

All experiments reported in this study, including the main comparisons and the additional robustness, ablation, and statistical analyses, initialize the image encoder from the original SAM ViT-B checkpoint (sam_vit_b_01ec64.pth); no released MedSAM checkpoint is used. The standard SAM image-encoder–prompt-encoder–mask-decoder interface is retained. In this study, “Full Fine-Tuning” (Full FT) denotes optimization of the complete SAM ViT-B image encoder and original mask decoder while the prompt encoder remains frozen. The experimental design distinguishes the unadapted checkpoint from task-trained settings. In HBF-BCER, pretrained image-encoder and prompt-encoder weights are frozen, whereas HBF, BCER, and decoder-adapter parameters are trainable. Table 1 defines each baseline and separates the contribution of the decoder adapter from that of the proposed encoder modules.

### 3.3. Historical Branch Fusion

**Figure 2:**
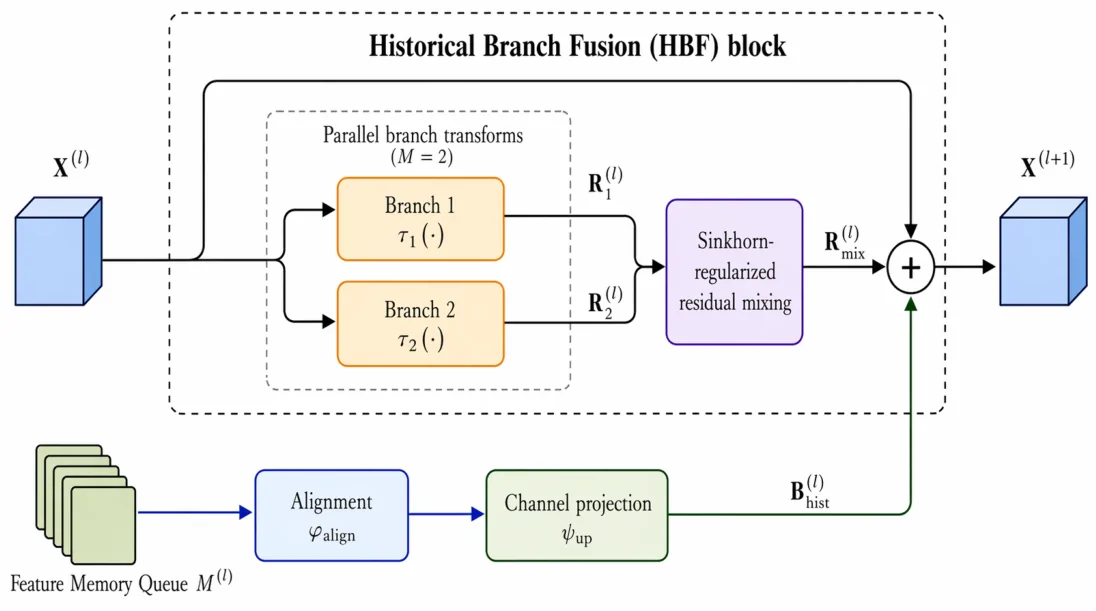
Historical Branch Fusion. A short feature-memory queue supplies a cross-layer historical bias, while Sinkhorn-normalized residual mixing and learnable branch aggregation refine the current block.

Figure 2 illustrates the internal structure of the HBF module.

For the *t*-th enhanced block, the standard transformer output $Y_{t}\in R^{B\times N\times C}$ is compressed to a bottleneck representation:

(6) $$D_{t}=\phi_{\mathrm{bn}}\left(Y_{t}\right),\;D_{t}\in R^{B\times N_{b}\times C_{b}},\;C_{b}=C/4.$$

The memory queue stores the previous *K* enhanced-block representations:

(7) $$M_{t}=\left\{D_{\max(1,t-K)},\ldots ,D_{t-1}\right\},\;K_{t}=\min\left(K,t-1\right).$$

when $K_{t}>0$, the historical summary and bias are

(8) $$\begin{array}{cc} H_{\mathrm{sum},t} & =\frac{1}{K_{t}}\sum_{j=\max(1,t-K)}^{t-1}\phi_{\mathrm{align}}\left(D_{j}\right), \end{array}$$

(9) $$\begin{array}{cc} B_{\mathrm{hist}}^{\left(l_{t}\right)} & =\psi_{\mathrm{up}}\left(H_{\mathrm{sum},t}\right). \end{array}$$

when no historical feature is available, the bias is zero.

Current bottleneck features are processed by *M* lightweight branches, $R_{t,m}=\tau_{m}\left(D_{t}\right)$. A nonnegative matrix $W_{t}\in R^{M\times M}$ is normalized by Sinkhorn iterations [28]:

(10) $$S_{t}=\mathrm{Sinkhorn}\left(W_{t}\right),\;\sum_{q}S_{t,mq}=1,\;\sum_{m}S_{t,mq}=1.$$

The branch-mixed response is

(11) $$\begin{array}{cc} {\bar{R}}_{t,m} & =\sum_{q=1}^{M}S_{t,mq}R_{t,q}, \end{array}$$

(12) $$\begin{array}{cc} \alpha_{t} & =\mathrm{Softmax}\left(a_{t}\right),\;R_{\mathrm{mix},t}=\sum_{m=1}^{M}\alpha_{t,m}{\bar{R}}_{t,m}, \end{array}$$

(13) $$\begin{array}{cc} B_{\mathrm{hbf}}^{\left(l_{t}\right)} & =\psi_{\mathrm{mix}}\left(R_{\mathrm{mix},t}\right). \end{array}$$

The learnable aggregation vector $\alpha_{t}$ prevents the doubly stochastic branch mixing from degenerating into a fixed uniform average.

### 3.4. Balanced Convolutional Expert Routing

**Figure 3:**
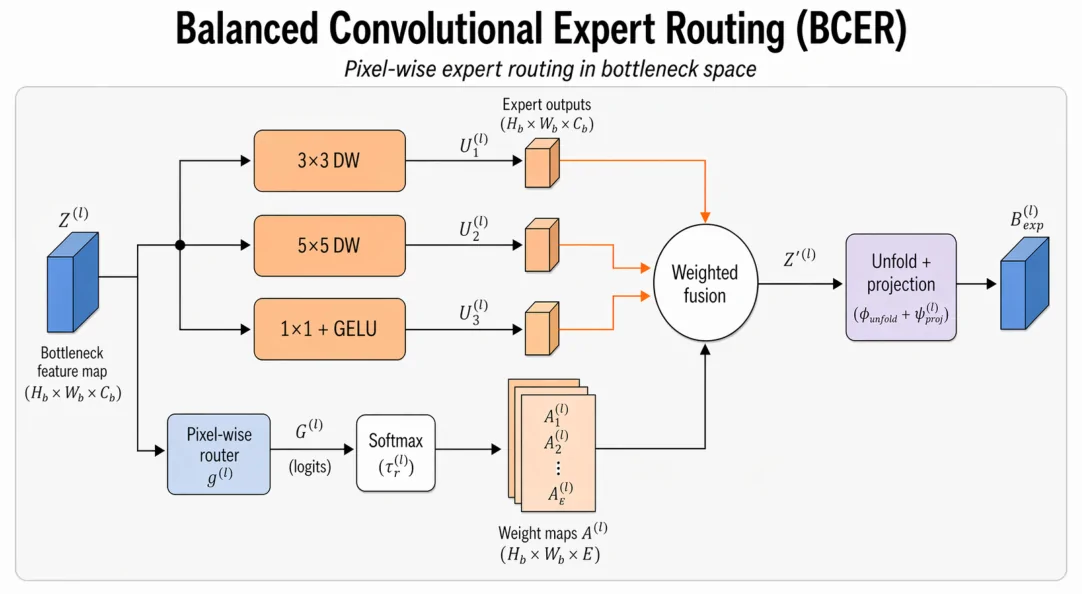
Balanced Convolutional Expert Routing. Pixel-wise soft weights combine three convolutional experts in bottleneck space; a load-balancing term discourages expert collapse. Ellipses in the weight-map stack denote the remaining expert weight maps.

Figure 3 illustrates the pixel-wise expert-routing mechanism of BCER.

After HBF refinement, tokens are folded into a bottleneck feature map:

(14) $$Z_{t}=\phi_{\mathrm{fold}}\left(\phi_{\mathrm{bn}}\left({\bar{X}}_{t}\right)\right)\in R^{B\times C_{b}\times H_{b}\times W_{b}}.$$

Three heterogeneous experts are used: depthwise–pointwise 3×3 and 5×5 convolutions and a 1×1 convolution with GELU. Their outputs are $U_{t,e}=f_{t,e}\left(Z_{t}\right)$. A pixel-wise router predicts logits $G_{t}=g_{t}\left(Z_{t}\right)$ and soft expert weights

(15) $$A_{t}=\mathrm{Softmax}\left(G_{t}/\tau_{r,t}\right)\in R^{B\times E\times H_{b}\times W_{b}}.$$

All experts are softly combined at each spatial position:

(16) $$Z_{t}^{\prime }=\sum_{e=1}^{E}A_{t,e}⊙U_{t,e},\;B_{\exp}^{\left(l_{t}\right)}=\psi_{\mathrm{proj}}^{\left(l_{t}\right)}\left(\phi_{\mathrm{unfold}}\left(Z_{t}^{\prime }\right)\right).$$

The mean usage of expert *e* is

(17) $$\mu_{t,e}=\frac{1}{BH_{b}W_{b}}\sum_{b,u,v}A_{t,b,e,u,v},$$

and the load-balancing loss is

(18) $$L_{\mathrm{bal},t}=\frac{1}{E}\sum_{e=1}^{E}{\left(\mu_{t,e}-\frac{1}{E}\right)}^{2}.$$

### 3.5. Optimization Objective

The segmentation objective combines Dice loss and binary cross-entropy [29,30]:

(19) $$L_{\mathrm{seg}}=\sum_{c=1}^{C_{m}}\omega_{c}\left[L_{\mathrm{Dice}}\left({\hat{M}}_{c},M_{c}\right)+L_{\mathrm{BCE}}\left({\hat{M}}_{c},M_{c}\right)\right].$$

The total objective is

(20) $$L_{\mathrm{total}}=L_{\mathrm{seg}}+\lambda_{\mathrm{bal}}\sum_{t=1}^{T}L_{\mathrm{bal},t},$$

where $T=|L_{\mathrm{enh}}|$ and $\lambda_{\mathrm{bal}}=0.01$.

## 4. Experiments and Results

### 4.1. Datasets, Quality Filtering, and Independent Evaluation Protocol

REFUGE2 is used for optic disc/cup segmentation. The official 800-image training set is divided into a fixed development split (90% training, 10% internal validation), while the 400-image official validation set is treated as a held-out evaluation set. The held-out set is not used for hyperparameter tuning or checkpoint selection. The internal-validation curve used for checkpoint selection is reported later in the convergence analysis rather than as held-out evaluation performance.

ISIC2016 follows its official training/test protocol. Ten percent of the official training set is reserved as an internal validation subset; the official test set is used only for final reporting.

TNMIX combines thyroid ultrasound images from the TNSCUI and DDTI sources. Preprocessing removes unreadable files, duplicate image–mask pairs, missing masks, empty foreground masks, and masks extending outside the image canvas. Images and masks are orientation-normalized, padded to preserve aspect ratio, and resized to 1024×1024. A fixed source-stratified split contains 3600 development images and 370 held-out evaluation images. Ten percent of the development pool is used for internal validation. Split assignment is fixed before model training, and no image identifier appears in more than one split. The split manifest, filtering script, and exclusion log are designated for public release with the final article.

### 4.2. Baselines and Implementation Details

All methods use the same image resolution, preprocessing, split, prompt coordinates, loss, optimizer, checkpoint-selection rule, and evaluation code. The backbone is SAM ViT-B. Consistent with the definition in Section 3.2, Full FT updates the complete image encoder and original mask decoder while the prompt encoder remains frozen. SAM + Adapter freezes the image and prompt encoders and trains only the decoder adapter. LoRA and DoRA use rank r=8, scaling α=16, dropout 0.05, and target the query/value projection matrices of the ViT image encoder; the same decoder adapter is retained so that differences are attributable to the encoder adaptation mechanism. HBF-only, BCER-only, and HBF-BCER use the same decoder adapter.

Adam is used with an initial learning rate of $10^{-4}$, zero weight decay, batch size 2, and StepLR with step size 10 and γ=0.5. Training lasts 40 epochs for REFUGE2, 20 epochs for ISIC2016, and 40 epochs for TNMIX on one NVIDIA RTX 3090 (24 GB). HBF uses K=3, M=2, and 20 Sinkhorn iterations. BCER uses E=3, an initial routing temperature of 1 clipped to [0.1,10], and $\lambda_{\mathrm{bal}}=0.01$. Each trainable method is repeated with three independent seeds (0, 1, and 2).

### 4.3. Prompt Generation and Robustness Protocol

Prompt coordinates are generated from ground-truth masks only for simulation; the ground-truth mask is not otherwise provided to the model. For the main REFUGE2 protocol, disc and cup are decoded in separate passes. Three positive foreground points are sampled for each target. During training, a box is additionally supplied with probability 2/3 and its overlap target is sampled from {0.50,0.75}. For ISIC2016 and TNMIX, three positive foreground points are sampled per target for the main comparison. A fixed prompt file is generated before evaluation and is shared by every method.

Prompt robustness is evaluated separately for REFUGE2 optic-cup segmentation in a one-interaction setting to avoid conflating it with the multi-point main protocol. The conditions are: mask-centroid point (accurate point), uniformly sampled foreground point, boundary-near interior point, point shifts of 2.5%, 5%, and 10% of the image diagonal, tight ground-truth bounding box, and boxes with 10%, 20%, or 30% coordinate jitter. The seed-0 checkpoint is used for every method; the seed index was fixed before robustness evaluation and was not selected according to robustness performance. Deterministic conditions contribute one prompt per held-out image. For stochastic conditions, ten prompt realizations per image are generated using a fixed prompt seed list. Dice is computed for each resulting image–prompt pair. The corresponding robustness results report the mean and standard deviation over all image–prompt observations.The dispersion therefore includes between-image heterogeneity and prompt-placement variability under a fixed checkpoint, but it does not include training-seed variability.

### 4.4. Metrics and Statistical Analysis

Dice, IoU, and HD95 are computed per image and then averaged [31,32,33,34]. Main tables report mean ± standard deviation across three training seeds. To assess paired differences independently of training variation, per-image Dice is first averaged across seeds for each method. The HBF-BCER-minus-baseline difference is then evaluated with a paired bootstrap (10,000 image-level resamples) and a two-sided Wilcoxon signed-rank test [35,36]. Holm adjustment is applied to the predefined family of four paired contrasts used for inferential analysis [37]. These four contrasts were included because matched per-image predictions for both methods were retained under identical held-out images and prompt coordinates, thereby permitting valid image-level paired analysis. They were not selected according to the observed effect sizes or *p*-values. Inferential statistical testing was not performed for the remaining method–target combinations; consequently, an unreported comparison means “not evaluated” rather than “no difference.” Statistical significance is defined as adjusted p<0.05.

### 4.5. Matched Main Results

Table 2 and Table 3 report only author-run, protocol-matched experiments. Literature values obtained under different splits or prompts are no longer used to support superiority claims.

HBF-BCER obtains the highest Dice for REFUGE2 cup and ISIC2016 and the lowest HD95 for all four reported targets. Full FT obtains the highest Dice for REFUGE2 disc (96.76% versus 96.69%) and TNMIX (92.74% versus 92.08%). For these two targets, HBF-BCER nevertheless has higher IoU and lower HD95. Across all four targets, HBF-BCER exceeds SAM + Adapter, LoRA, and DoRA on Dice. These results support an improved accuracy–boundary trade-off with substantially fewer trainable parameters, rather than universal superiority over full fine-tuning.

### 4.6. Paired Statistical Analysis

Table 4 reports the four paired per-image Dice contrasts defined by the availability of matched per-image predictions under identical images and prompts. After Holm correction, HBF-BCER significantly improves REFUGE2 cup relative to SAM + Adapter and LoRA and improves TNMIX relative to SAM + Adapter. The ISIC2016 difference relative to Full FT is not significant. Statistical significance was not evaluated for the remaining method–target combinations, and their omission should not be interpreted as evidence of no difference.

### 4.7. Prompt Robustness

Table 5 reports the one-interaction prompt-robustness experiment for REFUGE2 optic-cup segmentation. Within this target and protocol, all methods degrade as point displacement or box jitter increases. HBF-BCER achieves the highest mean Dice in every tested condition, but its margin over SAM + Adapter is modest. This pattern indicates improved representation quality for REFUGE2 optic-cup segmentation without eliminating the fundamental sensitivity of prompt-based segmentation to inaccurate user input. These findings are limited to this target and one-interaction protocol and should not be generalized to REFUGE2 disc, ISIC2016, TNMIX, or other interaction settings without additional experiments.

### 4.8. Internal HBF and BCER Ablations

Table 6 isolates the HBF design choices on REFUGE2. Increasing the memory length from zero to three improves overlap and HD95. Mean fusion underperforms Sinkhorn mixing, and removing the learnable aggregation weights slightly reduces accuracy. Table 7 shows that heterogeneous experts, pixel-wise routing, and load balancing each contribute. The complete BCER configuration performs best among the tested BCER-only variants.

### 4.9. Qualitative Analysis

**Figure 4:**
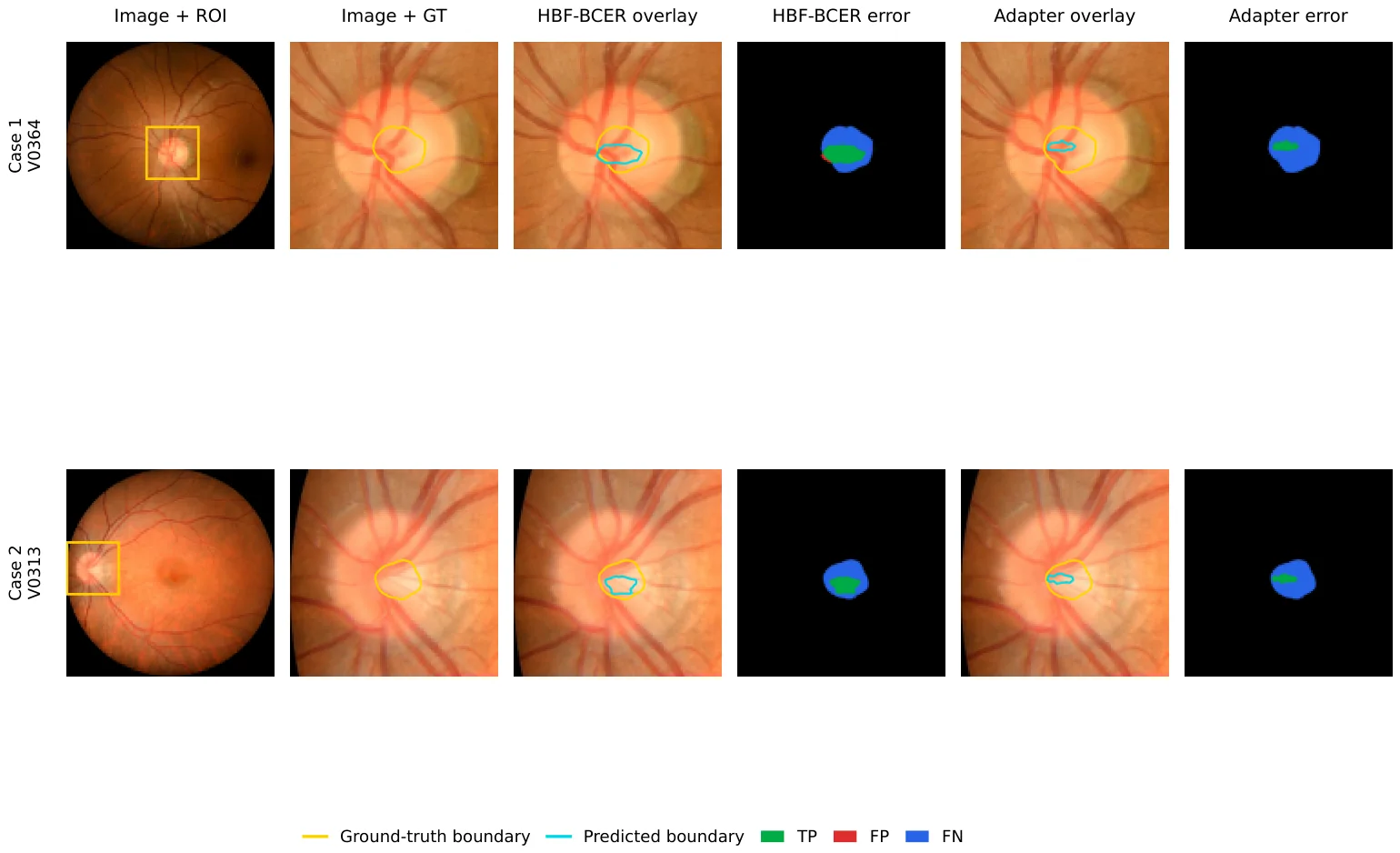
REFUGE2 qualitative comparison. Enlarged ROI overlays and error maps compare HBF-BCER with the matched SAM + Adapter control. Yellow/cyan contours indicate ground truth/prediction; green, red, and blue indicate TP, FP, and FN regions. The yellow rectangle in the first column indicates the region of interest (ROI) enlarged in the subsequent panels.

**Figure 5:**
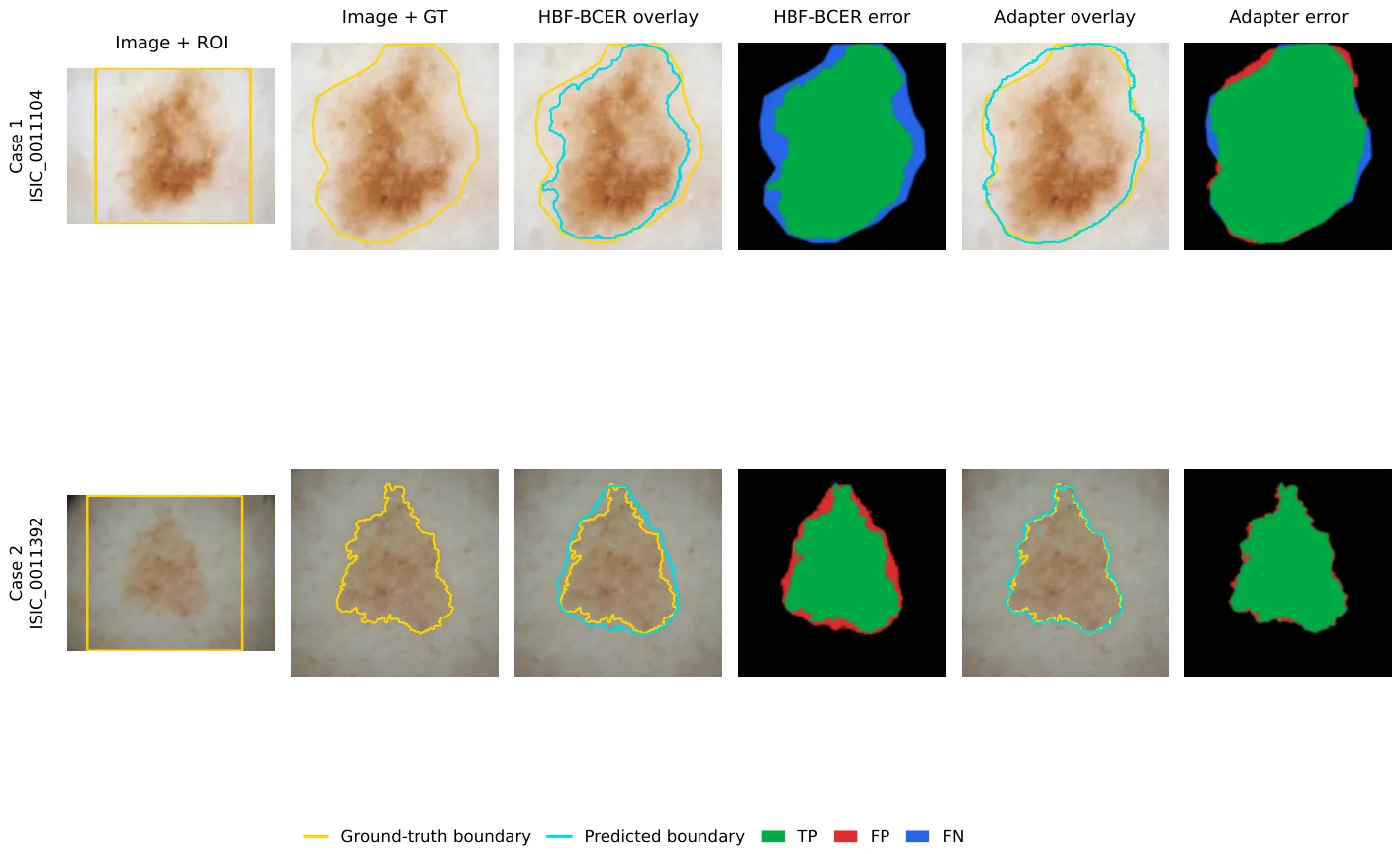
ISIC2016 qualitative comparison with enlarged boundary overlays and FP/FN error maps. The yellow rectangle in the first column indicates the region of interest (ROI) enlarged in the subsequent panels.

**Figure 6:**
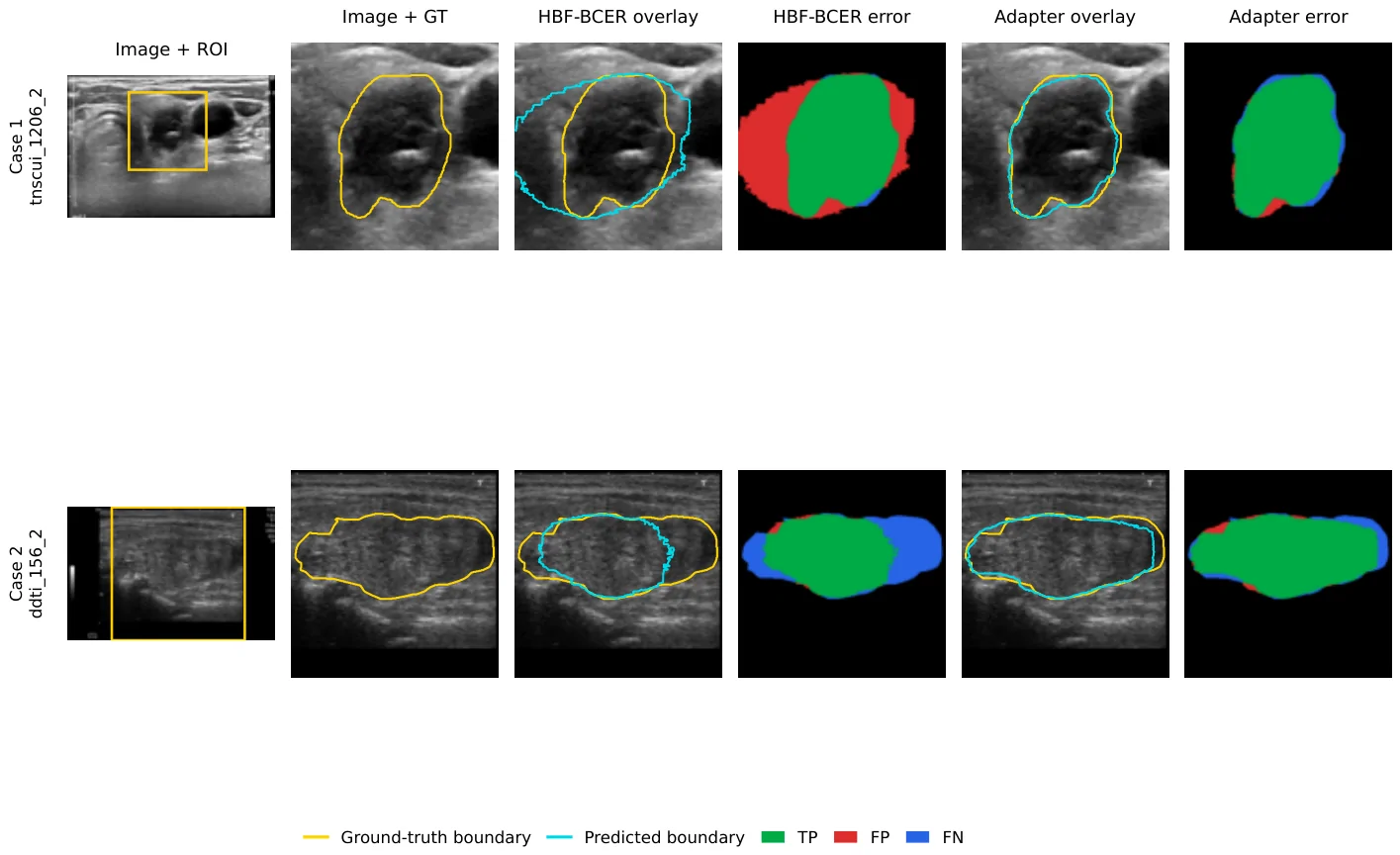
TNMIX qualitative comparison with enlarged thyroid-nodule overlays and FP/FN error maps. The yellow rectangle in the first column indicates the region of interest (ROI) enlarged in the subsequent panels.

**Figure 7:**
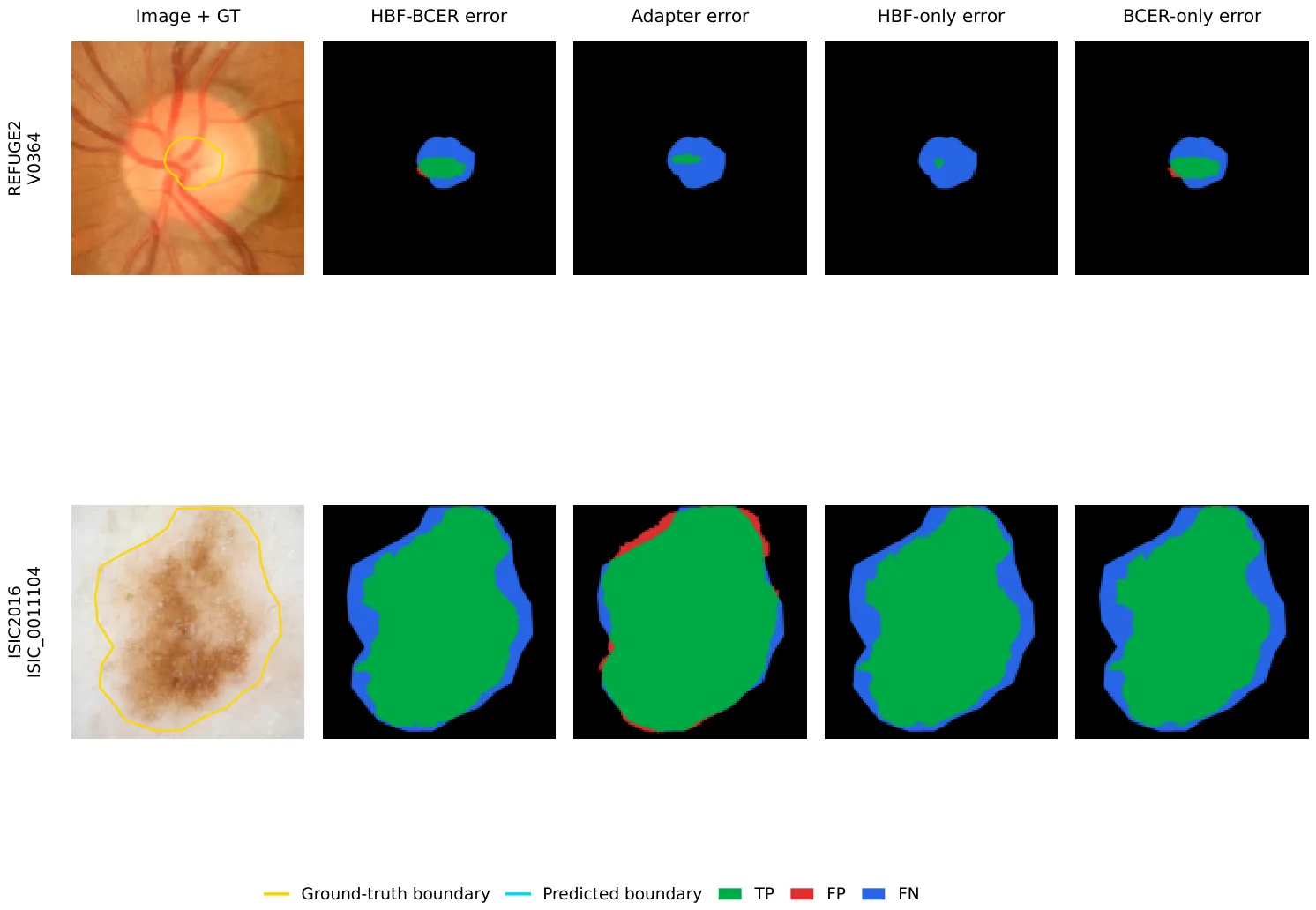
Cross-dataset error-map ablation comparing HBF-BCER, SAM + Adapter, HBF-only, and BCER-only variants.

Figure 4, Figure 5, Figure 6 and Figure 7 enlarge representative examples and display both contour overlays and color-coded error maps. Green denotes true positives, red false positives, and blue false negatives. On REFUGE2, the complete model better recovers the small cup region. On ISIC2016, it reduces boundary omissions and local leakage in irregular lesions. On TNMIX, it improves contour continuity under speckle noise, although difficult low-contrast cases still show both false-positive and false-negative regions.

### 4.10. Convergence and Efficiency

**Figure 8:**
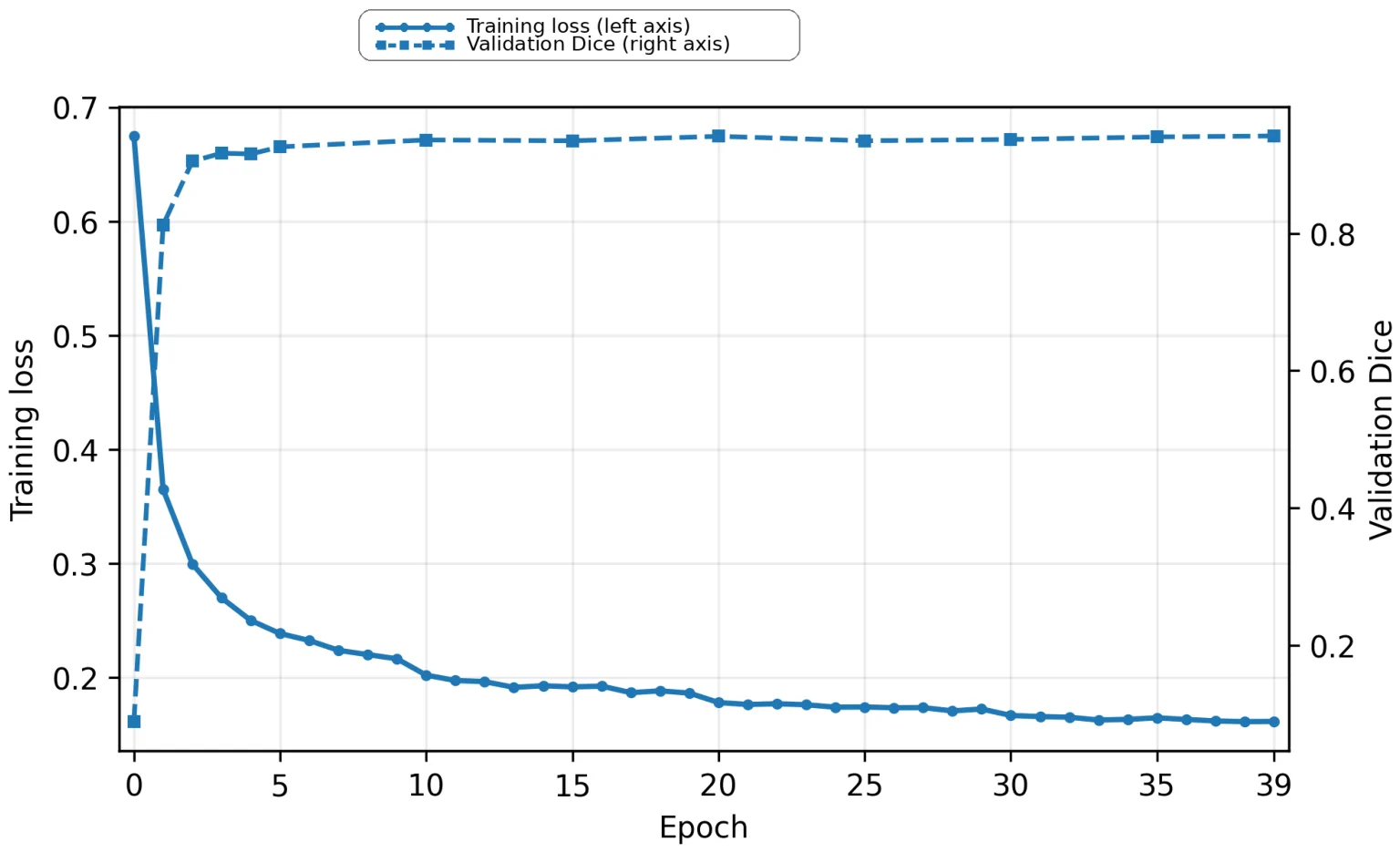
REFUGE2 convergence. The dashed curve shows internal-validation Dice used for checkpoint selection. The separate 400-image held-out evaluation set was not used for model selection.

Figure 8 shows REFUGE2 training loss and internal-validation Dice. The held-out evaluation set is not used to select the checkpoint. Table 8 reports the available efficiency measurements. HBF-BCER reduces trainable parameters by 81.5% relative to Full FT, but has more total parameters, FLOPs, and latency. It should therefore be described as parameter-efficient for training, not as a generally lightweight model.

Inference timing uses batch size 1, 1024×1024 inputs, PyTorch 2.8.0 (CUDA 12.8) evaluation mode, disabled gradients, 50 warm-up runs, 200 timed runs, and a CUDA synchronization before and after each timed forward pass on the same RTX 3090. Comparable peak GPU-memory and end-to-end wall-clock training measurements were not collected consistently for every baseline and are therefore not reported.

## 5. Discussion

The matched experiments clarify the sources of performance differences. The unadapted SAM ViT-B checkpoint performs poorly on the medical tasks, whereas full task fine-tuning and adapter-based training produce large improvements, showing that task adaptation accounts for a substantial part of the gain. HBF-BCER exceeds the decoder-adapter, LoRA, and DoRA controls on Dice for all four targets and achieves the lowest HD95 throughout. Full FT retains a slight Dice advantage for the REFUGE2 disc and a larger Dice advantage for TNMIX, while HBF-BCER has higher Dice for the REFUGE2 cup and ISIC2016. The reported paired analyses support significant gains for selected adapter and LoRA comparisons but do not establish universal superiority over Full FT.

HBF and BCER address complementary failure modes. HBF is most useful when small structures and early boundary evidence must remain accessible across deep encoder propagation. BCER adapts the local receptive field at each bottleneck pixel and is therefore relevant to heterogeneous lesion texture and ultrasound speckle. The internal ablations support the use of a three-block history, Sinkhorn mixing, learnable aggregation, heterogeneous experts, pixel-wise routing, and load balancing. The results do not imply that these mechanisms are fundamentally new principles; rather, the contribution is their implementation-aligned integration inside a prompt-preserving SAM ViT-B pipeline for medical segmentation.

The robustness experiment shows that model architecture alone cannot remove prompt sensitivity. All methods degrade substantially under 10% point displacement and 30% box jitter. HBF-BCER remains the strongest mean performer, but the margins are modest. In clinical use, iterative correction, uncertainty feedback, and explicit user studies remain necessary. The current simulated prompts quantify controlled robustness but should not be interpreted as a substitute for multiple human annotators.

**Figure 9:**
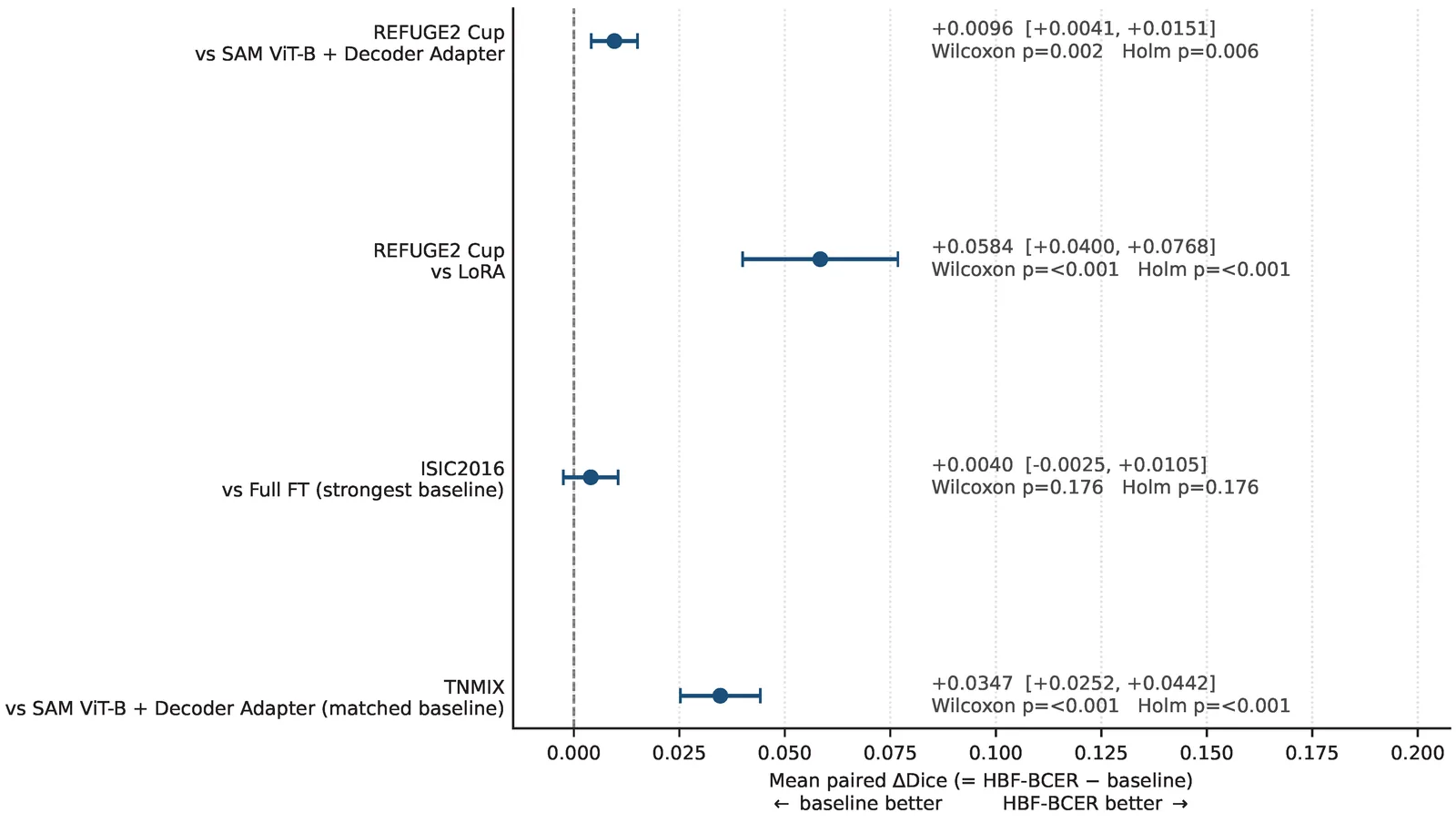
Paired per-image Dice differences for the four contrasts with retained matched per-image predictions. Points show mean HBF-BCER-minus-baseline differences and bars show 95% paired-bootstrap confidence intervals. Remaining method–target combinations were not subjected to inferential testing.

Figure 9 visualizes the four paired per-image Dice contrasts reported in Table 4.

### 5.1. Expected Extension to Volumetric Models

The present implementation is two-dimensional and does not claim volumetric validation. MedSAM2 and related SAM2-based systems introduce memory mechanisms for 3D images and videos [38,39,40]. A direct volumetric extension of HBF-BCER would require three changes. First, the HBF memory must distinguish encoder-depth history from inter-slice or temporal memory; unrestricted fusion could otherwise mix unrelated slices. Second, the 3×3 and 5×5 experts could be replaced by anisotropic 3×3×1, 3×3×3, or separable 3D experts to account for voxel spacing. Third, routing balance should be computed over volumetric locations while controlling GPU memory. HBF could complement the native SAM2 memory by preserving within-frame cross-layer structure, but interactions between the two memories must be evaluated rather than assumed. Volumetric CT/MRI experiments are therefore an important next step, not evidence supplied by the current 2D study.

### 5.2. Limitations

Several limitations remain. The training-seed count is three, which limits estimation of between-run variance. Prompt robustness is limited to REFUGE2 optic-cup segmentation, uses simulated interactions and fixed seed-0 checkpoints, and reports dispersion across image–prompt observations rather than across training seeds. TNMIX is a processed benchmark and requires public split manifests and filtering logs for complete reproducibility. Comparable peak GPU memory and wall-clock training time were not collected for all baselines and are therefore not reported. Finally, the current evaluation uses ViT-B and does not establish scaling behavior on larger backbones or MedSAM2.

## 6. Conclusions

HBF-BCER combines historical encoder-feature reuse with balanced pixel-wise convolutional expert weighting while preserving the standard point/box prompt interface. Under fixed held-out protocols and three training seeds, it achieves the highest Dice for the REFUGE2 cup and ISIC2016, while Full FT achieves the highest Dice for the REFUGE2 disc and TNMIX. HBF-BCER produces the lowest HD95 on all four targets and consistently exceeds the decoder-adapter, LoRA, and DoRA controls on Dice. The reported paired analyses show significant gains for REFUGE2 cup versus SAM + Adapter and LoRA and for TNMIX versus SAM + Adapter, while the ISIC2016 comparison with Full FT is not significant. HBF-BCER should therefore be viewed as a parameter-efficient training strategy with a favorable accuracy–boundary–trainable-parameter trade-off, not as a universally lightweight or uniformly superior model. Future work should include human prompt studies, public split/code release, larger multi-center datasets, complete resource logging, and volumetric validation with MedSAM2-style architectures.

## Figures and Tables

**Table 1:** Definition and trainable scope of all matched baselines and proposed variants. The checkpoint column identifies the actual pretrained initialization used in the experiments.

Setting	Checkpoint/Image Encoder	Prompt Encoder	Mask Decoder	HBF	BCER	Trainable Scope	ROLE
Original SAM ViT-B	SAM ViT-B, frozen	Frozen	Original, frozen	No	No	None	Unadapted reference
Image-encoder and mask-decoder full fine-tuning (Full FT)	SAM ViT-B, trainable	Frozen	Original, trainable	No	No	Complete image encoder + original mask decoder	Full image-encoder/mask-decoder task baseline
SAM + Adapter	SAM ViT-B, frozen	Frozen	Frozen + adapter	No	No	Decoder adapter	Adapter control
LoRA	SAM ViT-B + low-rank updates	Frozen	Frozen + adapter	No	No	LoRA + decoder adapter	Low-rank PEFT baseline
DoRA	SAM ViT-B + DoRA updates	Frozen	Frozen + adapter	No	No	DoRA + decoder adapter	Weight-decomposed PEFT baseline
HBF-only	SAM ViT-B, frozen	Frozen	Frozen + adapter	Yes	No	HBF + decoder adapter	HBF control
BCER-only	SAM ViT-B, frozen	Frozen	Frozen + adapter	No	Yes	BCER + decoder adapter	BCER control
HBF-BCER	SAM ViT-B, frozen	Frozen	Frozen + adapter	Yes	Yes	HBF + BCER + decoder adapter	Proposed framework

**Table 2:** Matched results on the held-out REFUGE2 evaluation set. Values are mean ± SD across three training seeds; Dice/IoU are percentages and HD95 is in pixels. Best values are shown in bold; ↓ indicates that lower values are better.

	Optic Disc			Optic Cup		
Method	Dice	IoU	HD95↓	Dice	IoU	HD95↓
Original SAM ViT-B	83.61 ± 0.48	72.17 ± 0.66	48.60 ± 2.10	33.48 ± 1.08	20.50 ± 0.86	83.20 ± 3.85
Full FT	**96.76 ± 0.10**	93.29 ± 0.18	6.21 ± 0.34	91.88 ± 0.24	85.21 ± 0.38	6.28 ± 0.31
SAM + Adapter	94.95 ± 0.18	90.49 ± 0.30	9.13 ± 0.52	91.28 ± 0.26	84.26 ± 0.42	6.84 ± 0.37
DoRA	94.20 ± 0.35	89.15 ± 0.49	10.30 ± 0.70	87.95 ± 0.42	79.08 ± 0.58	9.15 ± 0.55
LoRA	93.49 ± 0.31	88.02 ± 0.47	11.74 ± 0.88	86.40 ± 0.48	76.76 ± 0.60	9.90 ± 0.64
HBF-only	95.34 ± 0.20	91.18 ± 0.35	8.01 ± 0.43	91.72 ± 0.22	84.99 ± 0.36	6.43 ± 0.35
BCER-only	95.06 ± 0.22	90.67 ± 0.38	8.63 ± 0.48	90.94 ± 0.28	83.72 ± 0.44	7.09 ± 0.39
HBF-BCER	96.69 ± 0.12	**93.64 ± 0.21**	**5.69 ± 0.24**	**92.24 ± 0.18**	**85.85 ± 0.28**	**5.86 ± 0.23**

**Table 3:** Matched results on ISIC2016 and TNMIX held-out evaluation sets. Values are mean ± SD across three training seeds; Dice/IoU are percentages and HD95 is in pixels. Best values are shown in bold; ↓ indicates that lower values are better.

	ISIC2016			TNMIX		
Method	Dice	IoU	HD95↓	Dice	IoU	HD95↓
Original SAM ViT-B	24.39 ± 1.32	14.68 ± 0.95	138.50 ± 5.20	27.68 ± 1.15	16.85 ± 0.89	151.80 ± 6.10
Full FT	95.78 ± 0.20	**93.06 ± 0.36**	29.64 ± 1.12	**92.74 ± 0.17**	84.92 ± 0.28	34.85 ± 1.25
SAM + Adapter	95.60 ± 0.24	91.70 ± 0.41	31.27 ± 1.04	88.61 ± 0.28	79.60 ± 0.43	46.00 ± 1.86
DoRA	95.22 ± 0.35	91.16 ± 0.50	33.40 ± 1.80	91.30 ± 0.29	84.10 ± 0.42	36.90 ± 1.80
LoRA	94.89 ± 0.38	90.55 ± 0.55	35.78 ± 1.64	90.88 ± 0.34	83.45 ± 0.48	38.20 ± 2.10
HBF-only	95.27 ± 0.30	91.11 ± 0.46	34.13 ± 1.36	89.10 ± 0.27	80.45 ± 0.41	44.80 ± 1.62
BCER-only	95.26 ± 0.29	91.13 ± 0.45	33.75 ± 1.31	88.35 ± 0.30	79.28 ± 0.46	47.50 ± 1.71
HBF-BCER	**96.18 ± 0.31**	92.78 ± 0.54	**28.37 ± 0.92**	92.08 ± 0.19	**85.48 ± 0.31**	**34.20 ± 1.32**

**Table 4:** Paired statistical analysis of the four method–target contrasts for which matched per-image predictions were retained under identical held-out images and prompts. The contrasts were not selected according to observed effect size or statistical significance. Remaining method–target combinations were not subjected to inferential testing. Differences and confidence intervals are in Dice units. Holm-adjusted *p* values below 0.05 are shown in bold.

Dataset/Target	Comparison	Mean Difference	95% Bootstrap CI	Wilcoxon *p*	Holm-Adjusted *p*
REFUGE2 cup	HBF-BCER vs. SAM + Adapter	+0.0096	[0.0041,0.0151]	0.002	**0.006**
REFUGE2 cup	HBF-BCER vs. LoRA	+0.0584	[0.0400,0.0768]	<0.001	**<0.001**
ISIC2016	HBF-BCER vs. Full FT	+0.0040	[−0.0025,0.0105]	0.176	0.176
TNMIX	HBF-BCER vs. SAM + Adapter	+0.0347	[0.0252,0.0442]	<0.001	**<0.001**

**Table 5:** REFUGE2 optic-cup prompt robustness in the one-interaction protocol. Values are mean ± SD over all resulting image–prompt observations (%). Deterministic conditions use one prompt per image, whereas stochastic conditions use ten fixed realizations per image. Each method uses its seed-0 checkpoint; dispersion includes between-image heterogeneity and prompt-placement variability but not training-seed variability. The highest mean Dice within each prompt condition is shown in bold.

Prompt Condition	SAM + Adapter	LoRA	DoRA	HBF-BCER
Accurate point	70.86 ± 8.64	67.52 ± 9.41	68.79 ± 9.08	**71.84 ± 8.25**
Random foreground point	67.69 ± 9.62	64.47 ± 10.34	65.68 ± 9.95	**68.75 ± 9.21**
Boundary-near point	62.14 ± 10.86	59.02 ± 11.54	60.20 ± 11.18	**63.27 ± 10.42**
Point shift 2.5%	69.18 ± 9.17	65.96 ± 9.98	67.17 ± 9.57	**70.26 ± 8.79**
Point shift 5%	59.88 ± 11.24	56.81 ± 11.87	57.98 ± 11.53	**60.97 ± 10.89**
Point shift 10%	45.67 ± 12.68	43.19 ± 13.22	44.16 ± 12.95	**46.65 ± 12.34**
Tight box	77.50 ± 8.20	73.36 ± 9.30	74.67 ± 8.90	**78.32 ± 7.90**
Box jitter 10%	73.50 ± 8.90	69.57 ± 9.90	70.82 ± 9.60	**74.27 ± 8.60**
Box jitter 20%	66.60 ± 10.10	63.04 ± 10.90	64.17 ± 10.50	**67.30 ± 9.80**
Box jitter 30%	57.00 ± 11.60	53.95 ± 12.30	54.92 ± 12.00	**57.60 ± 11.30**

**Table 6:** Internal HBF ablation on REFUGE2. Dice/IoU are averages over disc and cup (%); HD95 is in pixels. Values are mean ± SD across three seeds. Best values are shown in bold; ↓ indicates that lower HD95 is better.

Variant	*K*	Historical Memory	Sinkhorn Mixing	Learnable α	Dice	IoU	HD95 ↓
No historical memory	0	No	N/A	N/A	93.16 ± 0.18	87.47 ± 0.29	7.60 ± 0.34
K=1	1	Yes	Yes	Yes	93.27 ± 0.17	87.62 ± 0.27	7.49 ± 0.31
K=2	2	Yes	Yes	Yes	93.41 ± 0.15	87.85 ± 0.24	7.33 ± 0.28
Mean fusion	3	Yes	No	No	93.32 ± 0.19	87.70 ± 0.30	7.43 ± 0.35
Sinkhorn without learnable α	3	Yes	Yes	No	93.40 ± 0.16	87.81 ± 0.25	7.36 ± 0.29
Complete HBF	3	Yes	Yes	Yes	**93.53 ± 0.13**	**88.09 ± 0.21**	**7.22 ± 0.24**

**Table 7:** Internal BCER ablation on REFUGE2. Dice/IoU are averages over disc and cup (%); HD95 is in pixels. Values are mean ± SD across three seeds. Best values are shown in bold; ↓ indicates that lower HD95 is better.

Variant	Experts	Routing	Balance Loss	Dice	IoU	HD95 ↓
Single 3×3 expert	3×3	None	No	92.58 ± 0.21	86.45 ± 0.32	8.65 ± 0.38
Uniform averaging	3×3/5×5/1×1	Uniform	No	92.68 ± 0.19	86.60 ± 0.29	8.45 ± 0.34
Image-level routing	Three experts	Global	Yes	92.77 ± 0.18	86.78 ± 0.28	8.28 ± 0.32
Pixel-wise routing without balance	Three experts	Pixel-wise	No	92.85 ± 0.17	86.92 ± 0.26	8.12 ± 0.30
3×3+5×5 experts	Two experts	Pixel-wise	Yes	92.91 ± 0.16	87.04 ± 0.25	7.98 ± 0.28
Complete BCER	Three experts	Pixel-wise	Yes	**93.00 ± 0.14**	**87.20 ± 0.22**	**7.86 ± 0.25**

**Table 8:** Efficiency comparison at 1024×1024 input resolution.

Method	Trainable Params	Total Params	FLOPs	Latency/Image	Interpretation
Full FT	93.74 M	93.74 M	976.91 G	125.71 ms	Full-parameter training
HBF-BCER	17.30 M	111.03 M	1039.36 G	144.62 ms	Parameter-efficient training

## Data Availability

REFUGE2 and ISIC2016 are available from their respective public repositories. The TNMIX split manifests, preprocessing and filtering rules, prompt-generation code, training configurations, evaluation scripts, and statistical-analysis scripts will be deposited in a public archival repository and made available with the published article.
